# Supplementary material for: Global impacts of marine heatwaves on coastal foundation species
Source: Nat Commun. 2024 Jun 13;15:5052. doi: 10.1038/s41467-024-49307-9 (PMC11176324; doi:10.1038/s41467-024-49307-9)
Supplement: Supplementary file 1 — Supplementary Information [file 41467_2024_49307_MOESM1_ESM.pdf]

Article to *Nature Communications*

Global impacts of marine heatwaves on coastal foundation species

Kathryn E. Smith[1*], Margot Aubin[1], Michael T. Burrows[2], Karen Filbee-Dexter[3,4], Alistair J. Hobday[5], Neil J. Holbrook[6,7], Nathan G. King[1], Pippa J. Moore[8], Alex Sen Gupta[9], Mads Thomsen[10,11], Thomas Wernberg[3,4], Edward Wilson[1], Dan A. Smale[1]

[1]Marine Biological Association of the United Kingdom, Plymouth, United Kingdom
[2]Scottish Association for Marine Science, Oban, United Kingdom
[3] Oceans Institute and School of Biological Sciences, University of Western Australia, Crawley, Western Australia, Australia
[4]Institute of Marine Research, His, Norway
[5]CSIRO Environment, Hobart, Tasmania, Australia
[6]Institute for Marine and Antarctic Studies, University of Tasmania, Hobart 7001 Tasmania, Australia.
[7]Australian Research Council Centre of Excellence for Climate Extremes, University of Tasmania, Hobart 7001 Tasmania, Australia.
[8]Dove Marine Laboratory, School of Natural and Environmental Sciences, Newcastle University, Newcastle-Upon-Tyne, United Kingdom
[9]Climate Change Research Centre, University of New South Wales, Sydney, New South Wales, Australia
[10]The Marine Ecology Research Group, Centre of Integrative Ecology, School of Biological Sciences, University of Canterbury, Christchurch, New Zealand
[11]Aarhus University, Department of Ecoscience, 4000 Roskilde, Denmark

*Corresponding author email: katsmi@mba.ac.uk

Supplementary Information Table 1. Generalized linear models carried out. Final model is described along with the factors removed due to high variance inflation score. All models were weighted by the number of sites that were averaged within each localised area (i.e. sites within 8 km of each other and sharing common environmental features).

Test	Model	Factors removed from the model due to high variance inflation score
Global invertebrate	Percent impacted ~ mean intensity + cumulative intensity + maximum absolute temperature + duration + ecoregion + point in range	Maximum intensity
Global macrophyte	Percent change ~ mean intensity + duration + ecoregion + point in range	Maximum intensity, cumulative intensity, maximum absolute temperature
Western Mediterranean	Percent MMEs ~ mean intensity + maximum intensity + cumulative intensity + maximum absolute temperature	Duration
Adriatic Sea	Percent MMEs ~ maximum intensity + duration	Mean intensity, cumulative intensity, maximum absolute temperature
Fiji	Percent bleaching ~ mean intensity + maximum intensity + cumulative intensity + maximum absolute temperature + duration	NA
Sunda Sea/Java Shelf	Percent bleaching ~ mean intensity + maximum intensity + cumulative intensity + maximum absolute temperature + duration	NA
Lesser Sunda	Percent bleaching ~ mean intensity + maximum absolute temperature + duration	Maximum intensity, cumulative intensity
Palawan / North Borneo	Percent bleaching ~ mean intensity + maximum intensity + maximum absolute temperature + duration	Cumulative intensity
Eastern Philippines	Percent bleaching ~ mean intensity + maximum intensity + maximum absolute temperature + duration	Cumulative intensity
Torres Strait and Northern GBR	Percent bleaching ~ mean intensity + maximum intensity + maximum absolute temperature + duration	Cumulative intensity
Central and Southern GBR	Percent bleaching ~ maximum intensity + cumulative intensity + maximum absolute temperature	Mean intensity, duration
Society Islands	Percent bleaching ~ mean intensity + maximum intensity + maximum absolute temperature + duration	Cumulative intensity
Western Caribbean	Percent bleaching ~ mean intensity + maximum intensity + maximum absolute temperature + duration	Cumulative intensity
Southwestern Caribbean	Percent bleaching ~ mean intensity + maximum intensity + cumulative intensity + maximum absolute temperature	Duration

Floridian	Percent bleaching ~ mean intensity + maximum intensity + cumulative intensity + maximum absolute temperature	Duration
Southern Caribbean	Percent bleaching ~ mean intensity + maximum intensity + cumulative intensity + maximum absolute temperature	Duration
Bahamian	Percent bleaching ~ mean intensity + maximum intensity + cumulative intensity + maximum absolute temperature	Duration
Greater Antilles	Percent bleaching ~ mean intensity + maximum intensity + cumulative intensity + maximum absolute temperature	Duration
Eastern Caribbean	Percent bleaching ~ mean intensity + cumulative intensity + maximum absolute temperature	Maximum intensity, duration
North and Central Red Sea	Percent bleaching ~ mean intensity + cumulative intensity + maximum absolute temperature	Maximum intensity, duration
East African Coral Coast	Percent bleaching ~ mean intensity + maximum intensity + cumulative intensity + maximum absolute temperature + duration	NA
Mascarene Islands	Percent bleaching ~ maximum intensity + duration	Mean intensity, cumulative intensity, maximum absolute temperature
Maldives	Percent bleaching ~ mean intensity + maximum intensity + cumulative intensity + maximum absolute temperature	Duration
Western Sumatra	Percent bleaching ~ mean intensity + duration	Maximum intensity, cumulative intensity, maximum absolute temperature
Tweed-Moreton	Percent change in cover of primary species ~ mean intensity + maximum intensity + maximum absolute temperature + duration	Cumulative intensity
Northern California	Change in density of primary species ~ mean intensity + cumulative intensity	Maximum intensity, maximum absolute temperature, duration
Southern California Bight	Change in density of primary species ~ mean intensity + maximum absolute temperature + duration	Maximum intensity, cumulative intensity
North Sea	Percent change in cover of primary species ~ maximum intensity + cumulative intensity + maximum absolute temperature	Mean intensity, duration
Cape Howe	Percent change in cover of primary species ~ mean intensity + cumulative intensity	Maximum intensity, maximum absolute temperature, duration
Bassian	Percent change in cover of primary species ~ mean intensity + maximum intensity + maximum absolute temperature	Cumulative intensity, duration

Supplementary Information Table 2. Results of General Linear Models comparing key marine heatwave characteristics to foundation species responses. GLMs for mass mortality events and bleaching used Quasibinomial error structure while those for seagrass and macroalgae used Gaussian error structure. Significant values are in bold. NA's in marine heatwave characteristics indicate characteristics that were removed due to collinearity. For location of ecoregions, see Spalding et al.[23]. AIC = Akaike Information Criterion.

Realm	Province	Ecoregion	Marine heatwave characteristics					df	AIC
			**Mean intensity (˚C)**	**Max intensity (˚C)**	**Cumulative intensity (˚C)**	**Max absolute temp (˚C)**	**Duration (days)**		
Mass mortality events in non-hard-coral habitat-forming invertebrates									
Temperate Northern Atlantic	Mediterranean Sea	Western Mediterranean	***0.016***	0.705	0.089	0.051	NA	59	NA
		Adriatic Sea	NA	0.172	NA	NA	***0.022***	11	NA
Bleaching in hard corals									
Central Indo-Pacific	Tropical Southwestern Pacific	Fiji	***>0.001***	***0.008***	0.178	0.276	0.474	65	NA
	Sunda Shelf	Sunda Sea/Java Shelf	***0.011***	***0.019***	0.921	***0.004***	0.470	31	NA
	Western Coral Triangle	Lesser Sunda	0.143	NA	NA	***0.024***	0.101	14	NA
		Palawan / North Borneo	0.140	0.939	NA	0.523	0.227	27	NA
		Eastern Philippines	0.255	0.385	NA	0.864	0.908	13	NA
	Northeast Australian Shelf	Torres Strait and Northern GBR	***0.033***	***0.021***	NA	0.134	0.621	17	NA
		Central and Southern GBR	NA	0.520	***0.012***	0.905	NA	12	NA
Eastern Indo-Pacific	Southeast Polynesia	Society Islands	0.836	0.415	NA	***0.013***	0.822	38	NA
		Western Caribbean	***0.034***	0.070	NA	***0.049***	0.253	39	NA

Tropical Atlantic	Tropical Northwestern Atlantic	Southwestern Caribbean	0.120	0.563	0.889	0.146	NA	16	NA
		Floridian	***0.042***	***0.008***	0.930	***<0.001***	NA	148	NA
		Southern Caribbean	0.236	0.056	0.657	***<0.001***	NA	18	NA
		Bahamian	0.772	0.249	0.964	0.149	NA	23	NA
		Greater Antilles	***0.037***	0.892	***<0.001***	***<0.001***	NA	95	NA
		Eastern Caribbean	0.612	NA	0.318	***<0.001***	NA	45	NA
Western Indo-Pacific	Red Sea and Gulf of Aden	North and Central Red Sea	***0.034***	NA	0.640	0.352	NA	26	NA
	Western Indian Ocean	East African Coral Coast	0.921	0.171	***0.002***	0.949	0.764	17	NA
		Mascarene Islands	NA	0.679	NA	NA	0.503	11	NA
	Central Indian Ocean Islands	Maldives	0.093	0.509	***0.023***	0.318	NA	24	NA
	Andaman	Western Sumatra	0.907	NA	NA	NA	0.065	9	NA
Change in primary species of seagrass									
Temperate Australasia	East Central Australian Shelf	Tweed-Moreton	**0.033**	0.057	NA	0.819	0.053	16	107.6
Change in primary species of macroalgae									
Temperate Northern Pacific	Cold Temperate Northeast Pacific	Northern California	***0.006***	NA	0.722	NA	NA	13	80.52
	Warm Temperate Northeast Pacific	Southern California Bight	***0.007***	NA	NA	0.534	0.684	27	138.04
Temperate Northern Atlantic	Northern European Seas	North Sea	NA	0.9148	***<0.001***	0.2032	NA	24	203.73
Temperate Australasia	Southeast Australian Shelf	Cape Howe	***0.0187***	NA	***0.0254***	NA	NA	16	135.11
		Bassian	0.462	0.294	NA	0.931	NA	20	140.99
