# Supplementary material for: Global impacts of marine heatwaves on coastal foundation species
Source: Nat Commun. 2024 Jun 13;15:5052. doi: 10.1038/s41467-024-49307-9 (PMC11176324; doi:10.1038/s41467-024-49307-9)
Supplement: Supplementary file 3 — Description of Additional Supplementary Files [file 41467_2024_49307_MOESM3_ESM.pdf]

Description of Additional Supplementary files

Supplementary Data 1. Average change in foundation species within an ecoregion. For habitat-forming invertebrates change represents proportion of the population impacted by mass mortality event. For seagrass and macroalgae change represents % change in cover or density of the primary species, as specified for each ecoregion. For corals change represents proportion of coral bleached. Where coral bleaching data were only available as ranges, mild (1-10%), moderate (11-50%) and severe (≥50%), the range was replaced with conservative values of 1%, 11% and 51%, respectively, to enable data to be incorporated. *All recorded responses the same. †Maximum range is based on conservative values as stated above.

Supplementary Data 2. Ranges of key marine heatwave characteristics identified in this study by ecoregion. * Only one replicate available. † More than one replicate taken from different localised areas with the same MHW signature. ‡ Data gathered across more than one foundation species type.
