# Supplementary material for: Global impacts of marine heatwaves on coastal foundation species
Source: Nat Commun. 2024 Jun 13;15:5052. doi: 10.1038/s41467-024-49307-9 (PMC11176324; doi:10.1038/s41467-024-49307-9)
Supplement: Supplementary file 6 — Reporting Summary [file 41467_2024_49307_MOESM6_ESM.pdf]

nature portfolio

Corresponding author(s): Kathryn Smith

Last updated by author(s): Oct 30, 2023

Reporting Summary

Nature Portfolio wishes to improve the reproducibility of the work that we publish. This form provides structure for consistency and transparency in reporting. For further information on Nature Portfolio policies, see our Editorial Policies and the Editorial Policy Checklist.

Statistics

For all statistical analyses, confirm that the following items are present in the figure legend, table legend, main text, or Methods section.

n/a	Confirmed	
☐	☒	The exact sample size (*n*) for each experimental group/condition, given as a discrete number and unit of measurement
☒	☐	A statement on whether measurements were taken from distinct samples or whether the same sample was measured repeatedly
☐	☒	The statistical test(s) used AND whether they are one- or two-sided<br>*Only common tests should be described solely by name; describe more complex techniques in the Methods section.*
☐	☒	A description of all covariates tested
☐	☒	A description of any assumptions or corrections, such as tests of normality and adjustment for multiple comparisons
☐	☒	A full description of the statistical parameters including central tendency (e.g. means) or other basic estimates (e.g. regression coefficient) AND variation (e.g. standard deviation) or associated estimates of uncertainty (e.g. confidence intervals)
☐	☒	For null hypothesis testing, the test statistic (e.g. *F*, *t*, *r*) with confidence intervals, effect sizes, degrees of freedom and *P* value noted<br>*Give P values as exact values whenever suitable.*
☒	☐	For Bayesian analysis, information on the choice of priors and Markov chain Monte Carlo settings
☒	☐	For hierarchical and complex designs, identification of the appropriate level for tests and full reporting of outcomes
☒	☐	Estimates of effect sizes (e.g. Cohen's *d*, Pearson's *r*), indicating how they were calculated

Our web collection on statistics for biologists contains articles on many of the points above.

Software and code

Policy information about availability of computer code

Data collection	Statistics for marine heatwaves were identified using the R package 'heatwaveR'
Data analysis	Data analyses were carried out in R Studio v. 2022.12.0+353 using the 'stats' and 'car' packages.

For manuscripts utilizing custom algorithms or software that are central to the research but not yet described in published literature, software must be made available to editors and reviewers. We strongly encourage code deposition in a community repository (e.g. GitHub). See the Nature Portfolio guidelines for submitting code & software for further information.

Data

Policy information about availability of data

All manuscripts must include a data availability statement. This statement should provide the following information, where applicable:

- Accession codes, unique identifiers, or web links for publicly available datasets
- A description of any restrictions on data availability
- For clinical datasets or third party data, please ensure that the statement adheres to our policy

The datasets used in this study are shown in Table 2. The processed data used in this study are available in the following figshare database: https://figshare.com/s/b7a4f926c746b2b9cc3a. Sea Surface Temperature data (dataset: ncdcOisst21Agg_LonPM180)used to determine the presence of marine heatwaves was downloaded from https://coastwatch.pfeg.noaa.gov/erddap/. Source data are provided with this paper for Figs 1-4. The data underlying Figures 5 and 6 can be found in the above figshare database. All datasets used in this study are freely available and the conditions of access were followed for each.

Research involving human participants, their data, or biological material

Policy information about studies with human participants or human data. See also policy information about sex, gender (identity/presentation), and sexual orientation and race, ethnicity and racism.

Reporting on sex and gender	NA
Reporting on race, ethnicity, or other socially relevant groupings	NA
Population characteristics	NA
Recruitment	NA
Ethics oversight	NA

Note that full information on the approval of the study protocol must also be provided in the manuscript.

Field-specific reporting

Please select the one below that is the best fit for your research. If you are not sure, read the appropriate sections before making your selection.

☐ Life sciences ☐ Behavioural & social sciences ☒ Ecological, evolutionary & environmental sciences

For a reference copy of the document with all sections, see nature.com/documents/nr-reporting-summary-flat.pdf

Ecological, evolutionary & environmental sciences study design

All studies must disclose on these points even when the disclosure is negative.

Study description	The study examines impacts of Marine Heatwaves on foundation species using a total of 2,208 observations across 85 marine ecoregions.
Research sample	Primary foundation species located in any global region where data were available. Our data cover macroalgae, seagrass, corals and other habitat-forming foundation species. The datasets used in the manuscript are shown in Table 2.
Sampling strategy	For comparison, density per unit area was used as the sampling unit for macroalgae and seagrass, proportion of corals bleached was used as the sampling unit for for corals, proportion of population impacted by mass mortality events was used as the sampling unit for other habitat-forming foundation species.
Data collection	Sampling strategies varied across species and citizen science programs, as did the person who recorded the data.
Timing and spatial scale	Time period: 1980-2021<br>Spatial scale: Global
Data exclusions	No data gathered below a depth of 10 m was used. This is because marine heatwaves are assessed using sea surface temperature data and consequently are most relevant to shallow water. For corals, only 'sheltered' reefs were included and 'exposed' reefs were removed. This is because there are often differences observed between bleaching rates on sheltered vs exposed shores and there were three times as many locations for sheltered reefs as there were for exposed reefs.
Reproducibility	The study involved the use of pre-existing data. All data are freely available, as is the R code for determining marine heatwaves.
Randomization	Observations were grouped by a) the foundation species type, and b) the marine ecoregion they were located in.
Blinding	Type of foundation species and, where possible, species, were allocated to each ecoregion, but the details of the specific location of the observation was kept blind for the analyses.

Did the study involve field work? ☐ Yes ☒ No

Reporting for specific materials, systems and methods

We require information from authors about some types of materials, experimental systems and methods used in many studies. Here, indicate whether each material, system or method listed is relevant to your study. If you are not sure if a list item applies to your research, read the appropriate section before selecting a response.

Materials & experimental systems

n/a	Involved in the study
☒	☐ Antibodies
☒	☐ Eukaryotic cell lines
☒	☐ Palaeontology and archaeology
☒	☐ Animals and other organisms
☒	☐ Clinical data
☒	☐ Dual use research of concern
☒	☐ Plants

Methods

n/a	Involved in the study
☒	☐ ChIP-seq
☒	☐ Flow cytometry
☒	☐ MRI-based neuroimaging

Plants

Seed stocks	NA
Novel plant genotypes	NA
Authentication	NA
